# Inside out: the role of nucleocytoplasmic transport in ALS and FTLD

**DOI:** 10.1007/s00401-016-1586-5

**Published:** 2016-06-06

**Authors:** Steven Boeynaems, Elke Bogaert, Philip Van Damme, Ludo Van Den Bosch

**Affiliations:** Department of Neurosciences, Experimental Neurology and Leuven Research Institute for Neuroscience and Disease (LIND), KU Leuven-University of Leuven, 3000 Leuven, Belgium; Laboratory of Neurobiology, Vesalius Research Center, VIB, Campus Gasthuisberg O&N4, PB912, Herestraat 49, 3000 Leuven, Belgium; Department of Neurology, University Hospitals Leuven, 3000 Leuven, Belgium

**Keywords:** Neurodegeneration, Aggregation, TDP-43, Nuclear transport, Nuclear pore, Importin, Exportin, Ran-GTP cycle

## Abstract

Neurodegenerative diseases are characterized by the presence of protein inclusions with a different protein content depending on the type of disease. Amyotrophic lateral sclerosis (ALS) and frontotemporal lobar degeneration (FTLD) are no exceptions to this common theme. In most ALS and FTLD cases, the predominant pathological species are RNA-binding proteins. Interestingly, these proteins are both depleted from their normal nuclear localization and aggregated in the cytoplasm. This key pathological feature has suggested a potential dual mechanism with both nuclear loss of function and cytoplasmic gain of function being at play. Yet, why and how this pathological cascade is initiated in most patients, and especially sporadic cases, is currently unresolved. Recent breakthroughs in *C9orf72* ALS/FTLD disease models point at a pivotal role for the nuclear transport system in toxicity. To address whether defects in nuclear transport are indeed implicated in the disease, we reviewed two decades of ALS/FTLD literature and combined this with bioinformatic analyses. We find that both RNA-binding proteins and nuclear transport factors are key players in ALS/FTLD pathology. Moreover, our analyses suggest that disturbances in nucleocytoplasmic transport play a crucial initiating role in the disease, by bridging both nuclear loss and cytoplasmic gain of functions. These findings highlight this process as a novel and promising therapeutic target for ALS and FTLD.

**Electronic supplementary material:**

The online version of this article (doi:10.1007/s00401-016-1586-5) contains supplementary material, which is available to authorized users.

## Introduction

Amyotrophic lateral sclerosis (ALS) and frontotemporal lobar degeneration (FTLD) are two devastating adult-onset neurodegenerative disorders. In ALS, motor neurons in the motor cortex, brainstem and spinal cord degenerate. This leads to motor problems, muscle weakness and paralysis. These motor impairments are progressive, and ALS is usually fatal within 3–5 years after diagnosis [114]. In FTLD, cortical neurons in the frontal and anteriotemporal cortex of the brain degenerate. FTLD patients can present with behavioral and/or personality changes or language problems [85]. In recent years, it has become increasingly clear that these seemingly unrelated diseases, ALS and FTLD, are the extremes of a disease spectrum. This idea originated from the clinical overlap in a number of patients presenting a mix of motor problems typical for ALS and behavioral changes characteristic of FTLD [114]. In the last decade, solid evidence has emerged for shared molecular mechanisms. Mutations in a set of genes can cause both diseases, and many ALS and FTLD patients share a similar pathology [68, 109].

For both ALS and FTLD, hereditary forms of the disease exist. In approximately 10 % of ALS patients and in 40 % of FTLD patients, the disease runs in the family. The neurodegeneration in these familial cases is caused by mutations in a heterogeneous set of genes (reviewed in Renton et al. [99]; Sieben et al. [109]). While the cause of the disease in sporadic cases is mostly unknown, the majority of patients present with similar neuropathological lesions: the RNA-binding protein TAR DNA-binding protein 43 (TDP-43) was identified as the major component of the ubiquitin-positive neuronal inclusion bodies observed in nearly all ALS patients (~97 %) and patients of the FTLD-TDP subtype (~45 %) [3, 68, 90]. The significance of this finding was consolidated by the subsequent discovery of mutations in the *TAR DNA*-*binding protein* gene (*TARDBP*), encoding TDP-43, in approximately 4 % of familial ALS patients [49, 99, 111] and rare FTLD cases [9]. This discovery has set the stage for a prime role for TDP-43 aggregation in ALS/FTLD pathogenesis. Besides TDP-43, another RNA-binding protein was found to be strongly implicated in the disease: mutations in *fused in sarcoma* (*FUS*) are found in ~5 % of ALS cases and rare cases of FTLD [60, 99, 123], and wild-type FUS protein aggregates are present in about 10 % of FTLD patients [89]. Moreover, patients carrying hexanucleotide repeat expansions in the *chromosome 9 open reading frame 72* gene (*C9orf72*), the most common genetic cause of ALS-FTLD [25, 100], have repeat RNA foci which sequester numerous RNA-binding proteins [20, 38, 64, 81]. Additionally, these patients also have dipeptide repeat peptides which interfere with RNA metabolism [61], and TDP-43 pathology [52]. These breakthroughs from the last decade have caused a paradigm shift in the ALS/FTLD field. The disease is no longer exclusively considered as a proteinopathy —a mere defect in protein folding— but is increasingly appreciated as a problem in ribostasis, or the conjoined misregulation of RNA-binding proteins and their responsive RNAs [98]. Despite these novel insights, the exact cause of the pathological aggregation of these RNA-binding proteins and their consequences remain elusive.

## RNA-binding proteins are strongly implicated in the pathology and genetics of ALS/FTLD

**Fig. 1:**
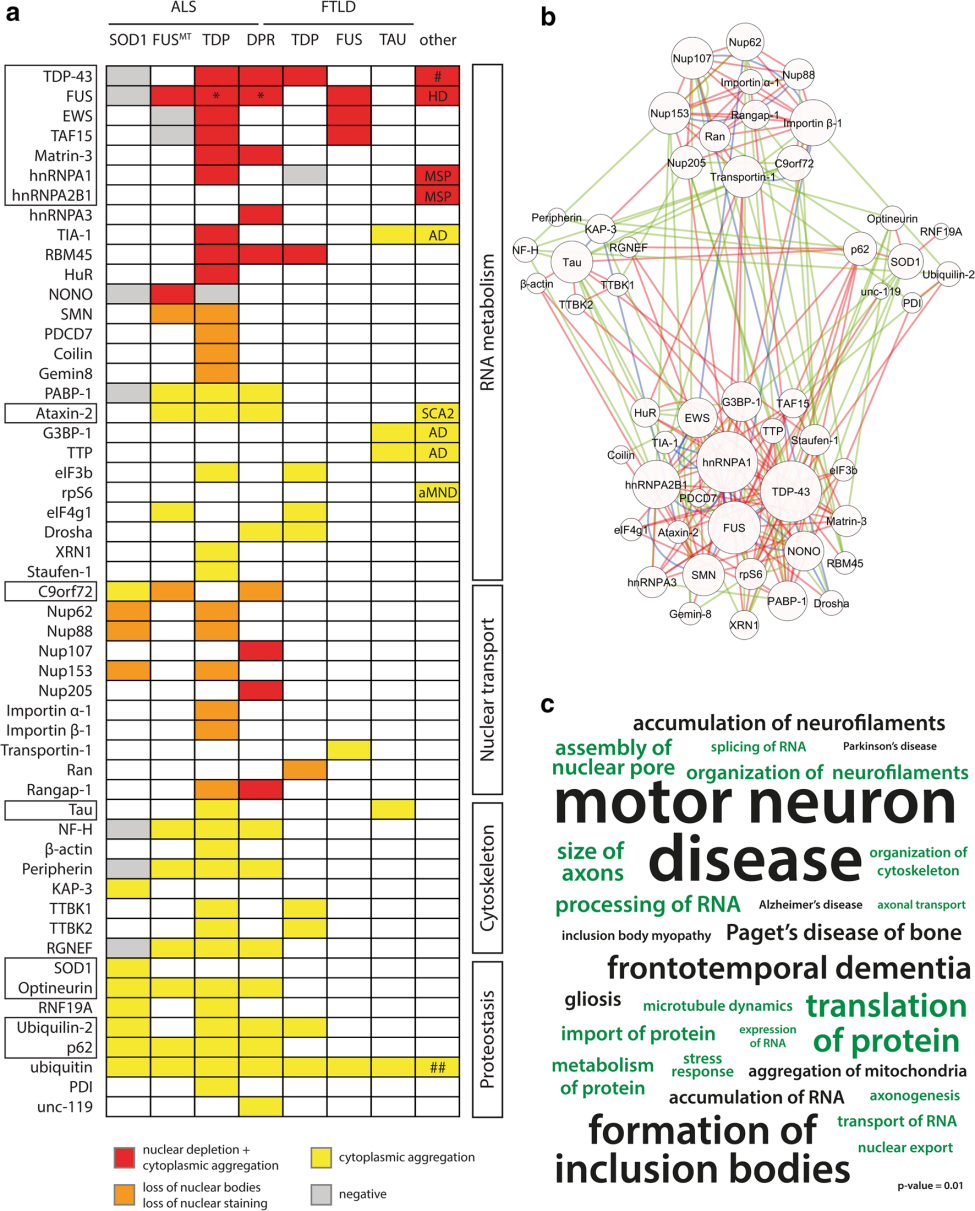
RNA-binding proteins are strongly implicated in ALS/FTLD pathology. **a** Schematic overview of protein mislocalization and aggregation in ALS/FTLD, as determined in post-mortem brain or spinal cord (muscle for MSP data). Patients are divided into different categories based on their pathology: SOD1, TDP-43, FUS, mutant FUS, Tau or DPR. DPR pathology is concurrent with RNA foci and TDP pathology. ALS/FTLD disease genes are *boxed*. Proteins can be grouped according to their function: ‘RNA metabolism’, ‘Nuclear transport’, ‘Cytoskeleton’ and ‘Proteostasis’. Misregulated RNA-binding proteins can be further divided into three classes based on their misregulation: I (*red*), II (*orange*) and III (*yellow*). *Asterisk* denotes debated findings; *hash* denotes occurrence in several neurodegenerative diseases; *double hash* denotes occurrence in all protein aggregation diseases. *AD* Alzheimer’s disease, *HD* Huntington’s disease, *MSP* multisystem proteinopathy, *SCA2* spinocerebellar ataxia type 2, *aMND* atypical motor neuron disease. **b** Network analysis using GeneMANIA indicates that all pathological proteins are highly interactive. Physical interactions are depicted in *red*, genetic in *green*, and colocalization in *blue*. Ubiquitin was not included in this network. **c** Word cloud depicting significantly overrepresented terms, as analyzed by ingenuity pathway analysis (IPA). Functional terms are *green*, disease and pathology terms *black*. Terms are scaled to the −log10(*p* value). References: TDP-43 [86], FUS [52, 87, 117], EWS [21, 87], TAF15 [22, 87], Matrin-3 [47], hnRNPA1 [42, 53, 88], hnRNPA2B1 [53], hnRNPA3 [81], TIA-1 [35, 70, 124], RBM45 [19], HuR [77], NONO [108], SMN [44, 122], PDCD7 [44], Coilin [44], Gemin-8 [122], PABP-1 [35, 75], ATX2 [29, 31], G3BP-1 [124], TTP [124], eIF3b [70], rpS6 [35], eIF4g [28], Drosha [96], XRN1 [125], Staufen-1 [125], C9orf72 [52, 133], Nup62 [58, 84], Nup88 [58], Nup107 [144], Nup153 [58], Nup205 [144], Importin α-1 [92], Importin β-1 [58, 84], Transportin-1 [91], Ran [126], Rangap-1 [133, 144], Tau [109, 137], NF-H [52, 131], β-actin [131], Peripherin [52, 131], KAP-3 [116], TTBK1 [66], TTBK2 [66], RGNEF [52], SOD1 [52, 128], Optineurin [16], RNF19A [41], Ubiquilin-2 [12], p62 [52], ubiquitin [101], PDI [6] and unc-119 [74]

To obtain a comprehensive view on protein misregulation in ALS and FTLD, we performed a systematic search of studies reporting protein mislocalization and/or aggregation in *post*-*mortem* patient material. PubMed search terms included ‘ALS’, ‘MND’, ‘FTLD’, ‘FTD’, ‘pathology’, ‘aggregation’, ‘aggregate’, ‘inclusion’ and ‘mislocalization’ (last search on 26/01/2016). Proteins were identified as misregulated when they were found aggregated in inclusion bodies or were mislocalized, based on immunohistochemistry or immunofluorescence in brain or spinal cord (muscle in multisystem proteinopathy patients). The results are graphically presented in Fig. 1a and show that at least 53 proteins are misregulated in patients [6, 12, 16, 19, 21, 22, 28, 29, 31, 35, 41, 42, 44, 47, 52, 53, 58, 66, 70, 74, 75, 77, 81, 84, 86–88, 91, 92, 96, 101, 108, 116, 117, 122, 124–126, 128, 131, 137]. This set of pathological proteins could be divided into four main groups based on functional annotations derived from UniProt and the literature. ‘RNA binding proteins’ constitute about 50 % of this list, highlighting their key role in the disease. The remaining proteins were involved in ‘Cytoskeleton’, ‘Proteostasis’ or ‘Nuclear transport’. While the first three categories are widely appreciated as important players in the disease [102], ‘Nuclear transport’ has received limited attention [26]. Using GeneMANIA software [127] and data from a recent large-scale interactomics study [39], we constructed an interaction network for the 53 identified misregulated proteins. Despite the large heterogeneity of the input set, the network included all proteins (Fig. 1b). This suggests that despite their different functions, they act together in common pathways. To identify these pathways, we performed enrichment analysis on the pathology protein set using Ingenuity Pathway Analysis (IPA^®^, QIAGEN, http://www.qiagen.com/ingenuity). Disease terms significantly enriched included ‘motor neuron disease’ and ‘frontotemporal dementia’, which can be expected given the origin of the input data set (Fig. 1c, Supplementary Data). Also, ‘Paget’s disease of bone’ and ‘inclusion body myopathy’ were found. This is also not surprising as it has become increasingly clear in the last few years that both these diseases share several pathological hallmarks and disease genes with ALS and FTLD [13, 118]. Consequently, all of these diseases are often classified as multisystem proteinopathies [118]. Pathology-related terms included ‘formation of inclusion bodies’ and other previously implicated abnormalities, such as ‘accumulation of neurofilaments’ [134] and ‘accumulation of mitochondria’ [73]. Functional terms included ‘translation of protein’ and ‘processing of RNA’, but also ‘transport of protein’ and ‘transport of RNA’ along with ‘axonal transport’. Indeed, the transport of ribonucleoprotein particles along the axon for local synaptic translation has become a prime pathological mechanism in ALS [2, 69], hereby bridging the gap between the earlier implicated cytoskeletal defects and the new interest in RNA metabolism.

We performed the same analysis for genes linked to ALS [99] and/or FTLD [109], and we found similar results (Fig. S1, Supplementary Data). This suggests that the identified actors and pathways, especially centering on RNA metabolism, are indeed implicated in the pathogenesis of these diseases.

## Dissecting RNA-binding protein pathology: what can we learn?

From the pathological and genetic findings, RNA-binding proteins emerge as a major class of actors in disease. Understanding the consequences but especially the cause of their misregulation could, hence, provide us with invaluable clues on the pathogenesis of ALS and FTLD.

We classified the implicated RNA-binding proteins in three major classes (see Fig. 1a) on the basis of their described pathological alterations. Firstly, proteins which under normal conditions mainly localize to the nucleus, but in the context of the disease mislocalize to the cytoplasm and aggregate (class I, red). Secondly, proteins which show a decreased number of nuclear bodies in disease (class II, orange). Thirdly, cytoplasmic proteins which also target the inclusion bodies seen in patients (class III, yellow).

Twelve of the affected RNA-binding proteins fall into class I. They include TDP-43 and FUS themselves, but also other related RNA-binding proteins which are mutated in rare familial cases, i.e., TATA-binding protein-associated factor 2N (TAF15) [22], EWS RNA-binding protein 1 (EWS) [21], Matrin-3 [47], and heterogeneous nuclear ribonucleoproteins A1 (hnRNPA1) [53] and A2B1 (hnRNPA2B1) [53]. Under normal conditions, these proteins are predominantly localized in the nucleus, where they serve essential functions in transcription and RNA processing. Hence, their nuclear depletion suggests a nuclear loss-of-function disease mechanism, besides the potential cytoplasmic gain of function associated with the inclusion bodies. Noteworthy in this regard are the different knockout and knockdown models generated which present with ALS-related phenotypes [43, 136] and gross transcriptome abnormalities [63]. Proteins in class II are as well depleted from their normal nuclear localization, hence, also suggesting a loss of function [44, 122]. Despite the nuclear predominance of class I proteins, they shuttle to the cytoplasm in a tightly regulated manner, where they control RNA transport, stability, decay and translation [62]. Interestingly, upon cellular stress, several of these RNA-binding proteins accumulate in large cytoplasmic ribonucleoprotein complexes called stress granules [7], and the formation of these stress granules is an essential step in the stress response. Proteins in class III are also known to localize to stress granules [7]. Given the strong similarities in protein content with the disease aggregates, stress granules have been suggested as potential stepping stones toward inclusion body formation [98]. Pathological aggregation hence could also perturb normal cytoplasmic function of these RNA-binding proteins or lead to a novel toxic gain of function.

Extensive efforts have been made to resolve the issue whether disease is caused by nuclear loss of function or cytoplasmic gain of function of the RNA-binding proteins. The available evidence so far suggests that both events are not mutually exclusive and could play an equally important role in the disease [68]. Identifying the pathways upstream of both cytoplasmic mislocalization and aggregation is of pivotal importance, as it could uncover targets for therapeutic intervention that prevent both nuclear loss-of-function and cytoplasmic gain-of-function mechanisms.

## Nuclear transport: bridge between loss and gain of function?

Given the nuclear depletion of several RNA-binding proteins in patients, impaired nuclear import has previously been suggested as a pathogenic mechanism, and moreover, as a key initiating event in pathogenesis [26]. Indeed, most pathogenic FUS mutations affect its nuclear localization sequence (NLS) and interfere with its proper nuclear targeting [28]. Compellingly, the nuclear/cytoplasmic ratio of different FUS mutants in vitro is inversely correlated with the age of disease onset in FUS-ALS patients [28]. In addition to alterations in its amino acid sequence, methylation of the FUS NLS also perturbs its nuclear targeting [27]. No NLS mutants for TDP-43 have been described in ALS or FTLD patients up to now. However, caspase-3 cleavage and alternative splicing in patients are known to generate an aggregation-prone C-terminal fragment, which lacks a functional NLS and, hence, is invisible for the nuclear transport machinery [135, 146].

Most mutations in TDP-43 [26], TAF15 [22], EWS [21], hnRNPA1 [53], hnRNPA2B1 [53] and several in FUS [26], specifically target their prion-like domains. These domains are disordered and contain amyloidogenic zipper motifs capable of inducing β-sheet formation and aggregation, similar to yeast prions [57]. Disease-causing mutations often target key conserved residues in these zippers [21, 22, 53], making these proteins more aggregation-prone. Once formed, aggregates of either mutant or wild-type RNA-binding proteins are able to seed prion-like aggregation of the remaining soluble pool. As these proteins shuttle constantly from nucleus to cytoplasm, such sequestration will trap these proteins in cytoplasmic aggregates and lead to a subsequent nuclear depletion.

**Fig. 2:**
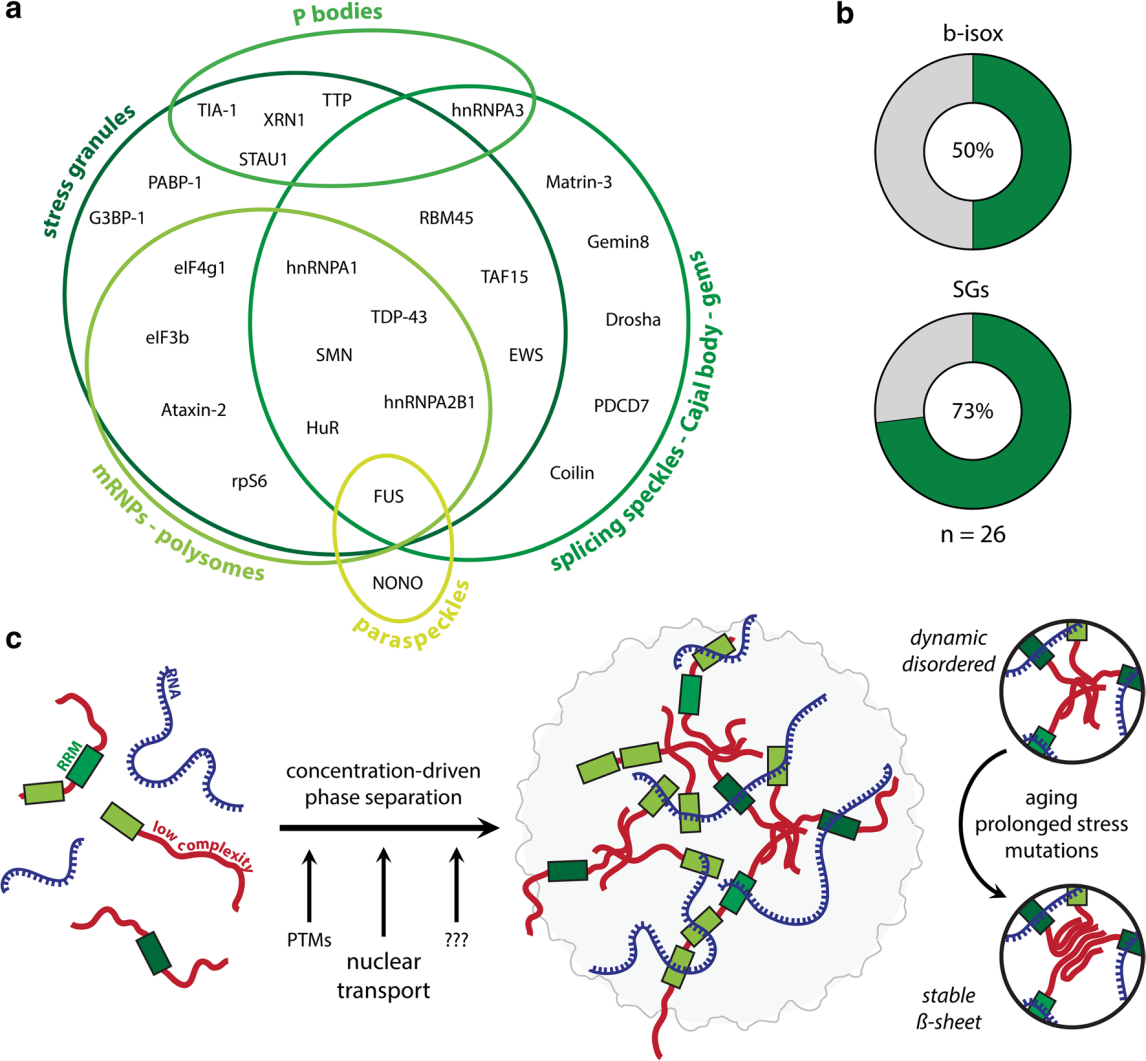
ALS/FTLD-related RNA-binding proteins undergo liquid–liquid phase transitions. **a** All RNA-binding proteins misregulated in ALS/FTLD are part of endogenous membrane-less organelles. **b** Several of these RNA-binding proteins are part of stress granules in vivo [7] or precipitate with β-isox in vitro [51] (*green* illustrates percent of total). **c** Scheme depicting RNA-binding protein phase transitions and the role of nuclear transport in this process. These phase transitions strongly depend on the local concentrations of the involved RNA-binding proteins. Disaggregases, such as VCP, and post-translational modifications (PTMs) also play a role in this process. Due to defects in the proper regulation of this process, e.g., aging and disease mutations, liquid-like stress granules can probably seed pathological aggregation

As mentioned above, cytoplasmic inclusion bodies have been proposed to arise from stress granules [26, 98]: more than 70 % of the pathological RNA-binding proteins that we identified are known stress granule components (Fig. 2a, b) [7]. This suggests that inclusion bodies could be seeded by stress granules or could arise from their improper clearance. Autophagy was shown to play a major role in this clearance, and interestingly, ALS/FTLD causing mutations in the autophagy protein valosin-containing protein (VCP) indeed perturb stress granule dissolution [13]. Recently, it was discovered that stress granules form by a process called phase transition. Similar to for example water, which can condense from vapor to fluid and freeze from fluid to solid, ALS- and FTLD- related RNA-binding proteins (e.g., TDP-43, hnRNPs and FUS) can undergo a similar phenomenon in vitro and in cell culture [15, 79, 83, 94] (Fig. 2c). Depending on specific in vitro conditions, such as concentration, salt and temperature, several RNA-binding proteins spontaneously demix from a watery solution and form liquid-like protein droplets. These droplets are highly reminiscent of cellular stress granules. Although disease-related mutations did not affect this liquid-like state, they promoted excessive β-sheet aggregation and transformed liquid droplets into solid aggregates [79, 83, 94]. As was the case for droplet formation, this maturation process was also strongly dependent on protein concentration. Partial maturation of the fluid-like interactions into more stable β-sheet structures seems to be inherent to stress granules in vivo [46, 132]. This illustrates the necessity of a tight control of stress granule dynamics to avoid excessive pathological aggregation.

Given the strong concentration dependence of stress granule dynamics, nucleocytoplasmic transport seems to be important in regulating this process. Indeed, inhibiting nuclear import of hnRNPs spontaneously induces the induction of stress granules [79]. On the other hand, overexpressing FUS NLS mutants [28] or removing the NLS sequences from TDP-43 [146] has similar effects. This shows that raising the cytoplasmic concentrations of these RNA-binding proteins is sufficient to induce stress granule formation. With respect to this, it is interesting to note that these proteins occur at endogenous intracellular concentrations which induce droplet formation in vitro (e.g., [hnRNPA1]_cell_ = 7.64 µM [39] > [hnRNPA1]_droplet_ = 0.5 µM [79]). This implies that cells must have evolved effective ways to regulate the initiation of such liquid-like phase transitions, more specifically, by controlling local concentrations through subcellular compartmentalization. Indeed, these proteins seem to withstand spontaneous phase separation at high concentrations in the nucleus for reasons currently unknown.

Nuclear transport factors themselves, such as importins and Ran, are well-established stress granule components [34, 72] and are known to regulate stress granule dynamics [17, 34, 138]. This suggests that these processes are indeed tightly linked and co-regulated. Of note, both FUS and TDP-43 control their cellular concentrations by means of autoregulation. High nuclear levels will result in a degradation of their own mRNAs through binding of the 3′UTR sequences [14, 147]. Pathogenic 3′UTR mutations which interfere with this process have been reported for both FUS and TDP-43 [36, 147]. This results in their overexpression which promotes aggregation. This crucial autoregulatory pathway once more stresses the need for proper nuclear import/export in the control of FUS and TDP-43 function.

Other membrane-less cellular compartments, e.g., the nucleolus or P granules, are also controlled by concentration-dependent phase transitions [10, 11]. Compellingly, all pathological RNA-binding proteins are found in such organelles (Fig. 2a), suggesting that this process is of major importance to the function of these RNA-binding proteins both in health and disease. For example, while a defect in nuclear import of class I proteins results in their cytoplasmic aggregation, a similar defect could underlie loss of nuclear bodies for class II proteins. In this case, lowered nuclear levels would lead to the dissolution of their nuclear bodies.

Nucleocytoplasmic transport bridges nuclear depletion and cytoplasmic aggregation, the two major pathological findings in ALS and FTLD. Besides this, nuclear transport factors are an important class of pathological proteins (Fig. 1a). Furthermore, mutations in *FUS* affect binding to its corresponding importin [28], and conversely, wild-type FUS inclusions sequester this specific importin [91]. All together, these findings advocate for a pivotal role of nucleocytoplasmic transport in the pathogenesis of ALS and FTLD.

## Nuclear transport factors are modifiers of ALS/FTLD disease models

We reviewed the literature on different ALS/FTLD models and their modifiers to find out whether there is support for a role for nucleocytoplasmic transport in the disease. Many research groups have harnessed the power of yeast and fly genetics to perform high-throughput genetic screens. Figure S2a gives an overview of the screens performed in ALS/FTLD models.

Fly ALS models of TDP-43, FUS and VAMP-associated protein B (VAPB) toxicity were used to identify genetic modifiers [24, 45, 142]. These modifiers included nuclear transport factors (Fig. S2a). Likewise, yeast genetic modifier screens for TDP-43 and FUS toxicity yielded modifiers in this process as well [4, 54, 113] (Fig. S2b). Despite the involvement of nuclear transport factors in these disease models, convincing evidence for a modifying role of these proteins has come from a recent work on *C9orf72*. Repeat expansions in the non-coding part of this gene result in the generation repeat RNAs [64, 129], which are translated by an ATG-independent mechanism into five dipeptide repeats (DPRs) [5, 80, 82, 148]. To investigate the underlying mechanisms of *C9orf72* ALS-FTLD, a genome-wide modifier screen in flies expressing an expanded GGGGCC repeat has been performed [32]. This led to the identification of numerous nuclear transport factors which were able to suppress or enhance the repeat RNA-induced rough eye phenotype [32] (Fig. S2a). Flies expressing the repeat expansion showed a defective nuclear mRNA export, which was reproduced in cortical neurons derived from induced pluripotent stem cells from *C9orf72* patients. As the model used in this study can display effects originating from both RNA and DPR toxicity, it remains unclear which of them causes the defects in nuclear transport. However, it has been suggested that in the current fly models with moderate repeat sizes, DPRs are the predominant mediators of neurodegeneration [78, 121].

The generation of codon-optimized ATG-dependent DPR expression constructs has allowed our laboratory and others to investigate protein toxicity independent from RNA toxicity in *C9orf72*. Expression of proline-arginine or glycine-arginine DPRs was toxic in yeast and induced rough eye phenotypes in fly [8, 48, 78, 121, 129]. Two genome-wide yeast screens [48] and a screen targeting nuclear transport in fly [8] uncovered similar modifier genes, which also showed partial overlap with the modifiers from the GGGGCC screen (Fig. S2a). These findings suggest an important role of DPRs in the nucleocytoplasmic transport defects observed in these models. Another recent study concludes that direct RNA toxicity is involved in these defects as well, through an aberrant interaction of GGGGCC RNA with Ran GTPase-activating protein 1 (RanGAP1) [144], a key regulator of the energy gradient driving nucleocytoplasmic transport (Fig. S2a). Interestingly, RanGAP1 was also found trapped in GA aggregates in mouse brain [145]. Lastly, the *C9orf72* loss-of-function hypothesis, due to lowered transcription levels, has not yet been ruled out. Recent reports suggest that the protein itself could operate in autophagy [105] and stress granule assembly [71], but also in nucleocytoplasmic transport [133].

All these suggested mechanisms in *C9orf72* pathogenesis are not mutually exclusive, and future work is required to untangle their relative contributions to the disease (reviewed in [37]). However, all of them seem to point toward nucleocytoplasmic transport as a key player in *C9orf72* pathogenesis, confirming the previously anticipated role for this process in ALS and FTLD [26].

## Nuclear transport defects are implicated in ALS and FTLD

The recent developments in the C9orf72 field point at key pathways in nucleocytoplasmic transport. These findings could explain the mislocalization and aggregation of TDP-43 and other RNA-binding proteins in patients carrying a repeat expansion. Yet, why wild-type proteins mislocalize in other types of the disease, and especially in sporadic patients, remains largely enigmatic.

ALS and FTLD are typically adult-onset disorders, suggesting that aging and its related processes are crucial to induce the disease. Interestingly, the efficiency and selectivity of nucleocytoplasmic transport deteriorates significantly during aging [23]. This can be largely attributed to the fact that several nuclear pore proteins are among the most long-lived proteins in post-mitotic neurons [103]. Postmitotic cells are unable to repair damaged nuclear pores, which become leaky during aging. Oxidative stress, a well-known hallmark of aging and implicated in several neurodegenerative disorders [65], is known to cause such damage to the nuclear pore [23, 140], but also to other components of the nuclear transport machinery [18, 59]. Several nuclear transport factors themselves have already been found misregulated in *post*-*mortem* ALS and FTLD patient material [58, 84, 92, 126, 144] (Fig. 1a). However, the precise reason for this mislocalization has remained elusive. A recent landmark study concludes that defects in proteostasis could be involved in this process [130]. It was found that cytoplasmic aggregation of synthetic amyloidogenic proteins, as well as disease relevant proteins, such as TDP-43 fragments, did cause mislocalization and aggregation of nuclear pore subunits and other nuclear transport factors. This resulted in defects in protein import and also in mRNA export. Interestingly, mice expressing mutant superoxide dismutase 1 (SOD1) showed misregulation of different nuclear pore components and import factors [143]. Additionally, aggregates of mutant VAPB [120] or TDP-43 [107] also affected the solubility of these proteins (Fig. S2b). Of note, wild-type VAPB itself seems to be important in nuclear pore assembly [120]. Lastly, wild-type FUS aggregates sequester transportin-1 and coaggregate with other cargoes in FTLD-FUS cases [91].

Besides protein aggregation, defects in RNA metabolism are considered to be a cornerstone of ALS/FTLD pathogenesis [68]. TDP-43 itself regulates numerous nuclear transport genes [106, 126]. Consequently, TDP-43 deregulation could initiate a positive feedback loop by perturbing nuclear transport. TDP-43 knockdown in cell lines led to depletion and mislocalization of several nuclear transport factors [112] (Fig. S2b). Also, TDP-43 mislocalized in an FTLD mouse model, and this was accompanied by nuclear depletion of Ran GTPase (Ran) [126]. TDP-43 was shown to regulate Ran expression, and loss of TDP-43 function resulted in lower Ran levels. Moreover, overexpression of Ran was able to rescue neurodegeneration and TDP-43 mislocalization in cortical neurons derived from this mouse model [126]. This latter finding, together with the recent *C9orf72* reports [32, 48, 144], suggests that nucleocytoplasmic transport could be an interesting therapeutic strategy.

Despite the importance of nuclear transport proteins in the pathology of ALS/FTLD, this group of genes has not been strongly implicated in the genetics of the disease so far. A notable exception is *GLE1 RNA export mediator* (*Gle1*). Mutations in *Gle1* were recently linked to ALS cases [50]. *Gle1* knockdown did induce motor neuron defects in embryonic zebrafish, which could be rescued by wild type but not by mutant *Gle1*. Besides functioning in mRNA export, Gle1 has been also shown to regulate as well stress granule dynamics [1]. This genetic link of ALS with an established mRNA export factor once more illustrates the potential importance of this process in the disease. As indicated before, the C9orf72 protein is also a suspected nuclear transport factor [133], which could further increase the genetic evidence for a role of nuclear transport in the disease.

## Can nuclear transport defects initiate disease?

**Fig. 3:**
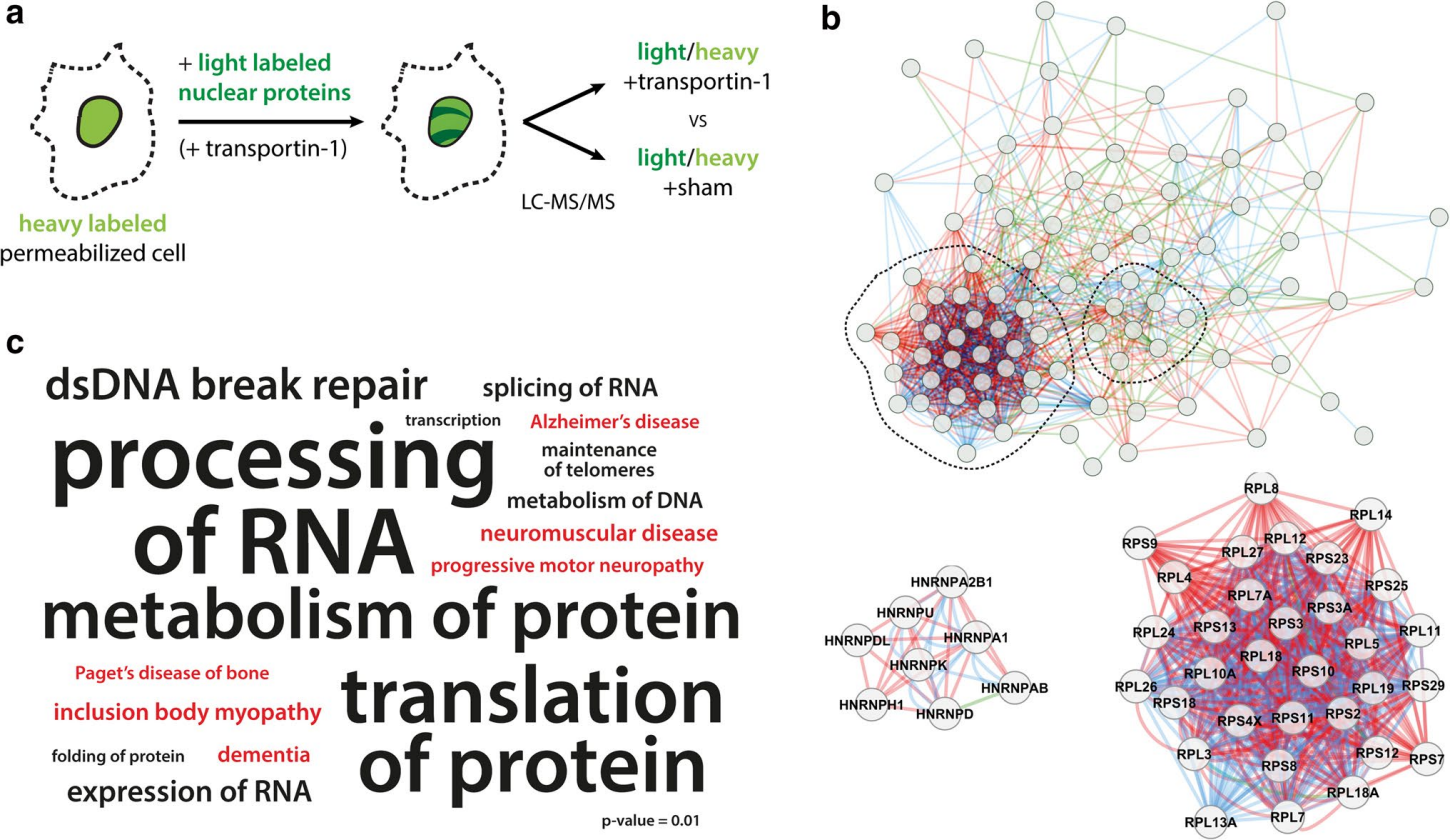
Nuclear transport cargoes are strongly implicated in ALS/FTLD and related pathways. We analyzed transportin-1 and exportin-1 cargo lists, which were experimentally validated. **a** Scheme of experimental setup for determining transportin-1 cargoes [55]. **b** These cargo proteins form a dense interaction network. Two subnetworks, the ribosome and hnRNP family, are highlighted. *Red* depicts physical interactions, *green* genetic, and *blue* colocalization. **c** Word cloud of significantly overrepresented terms, as analyzed by ingenuity pathway analysis (IPA). Functional terms are *black*, and disease terms *red*. Terms are scaled to the −log10 (*p* value)

Nucleocytoplasmic transport involves protein import and export, mediated by importins and exportins, respectively. Numerous different import and export factors exist, but the most important ones include transportin-1, the importin α2/β1 complex and exportin-1. Interestingly, these different import factors were recently identified in different genetic screens for modifiers of *C9orf72* toxicity [8, 32, 48, 144], as discussed above. Using quantitative mass spectrometry in combination with in vitro import assays, two studies aimed to identify cargoes dependent on transportin-1 or importin α2/β1 for their nuclear targeting (Fig. 3a) [55, 56]. In each case, about 80 high-confidence cargoes were found using stringent cut-off criteria. We used these proteins to reconstruct interaction networks for both import pathway clients. Figure 3b shows the highly interactive network of transportin-1 cargoes, suggesting that all these proteins share similar functions. Two subnetworks drew our interest: firstly, ribosomal proteins are one of the main clients of nuclear import, since after cytoplasmic translation, these proteins need to shuttle back to the nucleolus for ribosomal subunit assembly. Of note, nucleolar stress and a defective ribosomal biogenesis have been recently implicated in ALS/FTLD models [38, 48, 61, 115, 129]. Secondly, several hnRNP family members were also detected among the transportin-1 cargoes. This family of RNA-binding proteins has been increasingly implicated in ALS/FTLD in the last years [42, 81], and mutations in some of them are rare causes of the disease [53]. Enrichment analysis uncovered strong associations with ALS/FTLD-related pathways for the entire cargo set. Significantly enriched terms especially centered on RNA metabolism and protein translation (Fig. 3c, Supplementary Data). Moreover, when looking at enriched disease terms, we found that these sets of proteins were associated with ‘neuromuscular disease’ and ‘dementia’. Also, the related proteinopathies ‘Paget’s disease of bone’ and ‘inclusion body myopathy’ resulted from this analysis. Network and enrichment analysis of the importin α2/β1 dataset gave similar results as for transportin-1 (Supplementary Data).

Nuclear export mediated by exportin-1 can be specifically inhibited by leptomycin B [119]. Using quantitative mass spectrometry, proteins affected in their subcellular distribution by this compound have been identified [119]. Whereas most proteins were unaffected, just over 100 proteins showed a significant depletion from the cytoplasm and/or accumulation in the nucleus. When analyzing this set for enriched functional and disease terms, similar pathways were found as for the importin cargo sets (Supplementary Data). Interestingly, nuclear transport factors themselves were also misregulated by leptomycin B treatment. Indeed, nucleocytoplasmic transport is dependent on the continuous translocation of different effectors back and forth in and out of the nucleus to maintain their appropriate cellular distribution, e.g., importins need to go back to the cytoplasm for binding new NLS cargoes. Hence, disrupting one aspect of this process will have widespread effects on both import and export.

Our analyses of nuclear transport cargo sets illustrate that problems in nucleocytoplasmic transport will affect processes implicated in the disease, especially centering on RNA metabolism. Moreover, the fact that these cargo sets show a strong enrichment for proteins already implicated in ALS/FTLD and related disorders, suggests that defects in nuclear transport could initiate important pathogenic cascades.

## Why are neurons vulnerable for nuclear transport defects?

Nucleocytoplasmic transport is an essential process in cellular organization and functioning. So, if defects in nuclear transport are a cause of ALS/FTLD, why is degeneration largely restricted to specific neuronal populations? Explaining this discrepancy between general pathogenic pathways and selective cell death has been puzzling the neurodegeneration field for years, and applies to numerous established disease mechanisms, e.g., excitotoxicity, mitochondrial damage or protein aggregation [104].

A potential reason why nucleocytoplasmic transport defects could especially harm neurons can be found in their extreme cellular organization: neurons are the longest cells in the human body, e.g., motor neurons can reach lengths of up to 1 m in adults. This means that the synapse, the site of action, is located on a phenomenal distance from the nucleus. Synapses are, for their proper functioning, dependent on local translation and, hence, RNA transport along the axon. Numerous ALS/FTLD-associated genes are implicated exactly in this process [2, 69]. Also, nuclear transport factors themselves are found locally in the axon and at the axon terminals where they play a crucial role in this long-distance communication [95, 141]. As discussed above, the post-mitotic nature of neurons predisposes them as well to age-related disturbances in nucleocytoplasmic transport [23, 103].

On the other hand, the central nervous system is a hot spot for alternative splicing events [97, 139]. Brain alternative splicing events are also more conserved during evolution than in other tissues [76], illustrating the importance of this process in normal brain functioning [97]. Such tissue-specific splicing events seem to fine-tune protein–protein interaction networks according to the specific need of the cell type [30]. Both ALS- and/or FTLD-linked genes and pathological proteins were significantly enriched for splicing factors (‘splicing of RNA’; Benjamini–Hochberg corrected *p* value = 1.21E−03 and 1.84E−03). Defects in splicing are also a common theme in neurological diseases [67]. Maintaining proper nuclear levels of splicing factors, hence, is key to neuronal functioning.

## Conclusions

Landmark discoveries in the last decade have vastly expanded our knowledge on ALS and FTLD. The involvement of RNA-binding proteins has moved the field from viewing the diseases as exclusive proteinopathies to a more holistic view of ALS/FTLD as a problem in ribostasis, linking RNA-binding protein aggregation to problems in RNA metabolism. Mutations in numerous genes encoding such proteins strengthened this hypothesis, and finally, the discovery of the *C9orf72* expansion consolidated the importance of RNA metabolism in the disease. However, key issues remain to be addressed. Especially, the exact underpinnings of RNA-binding protein mislocalization proved elusive. NLS mutations were identified in FUS, but why wild-type proteins mislocalize was unknown. Only very recently, the identification of nuclear transport genes as modifiers of *C9orf72* disease models shifted the focus of the field explicitly to nucleocytoplasmic transport.

**Fig. 4:**
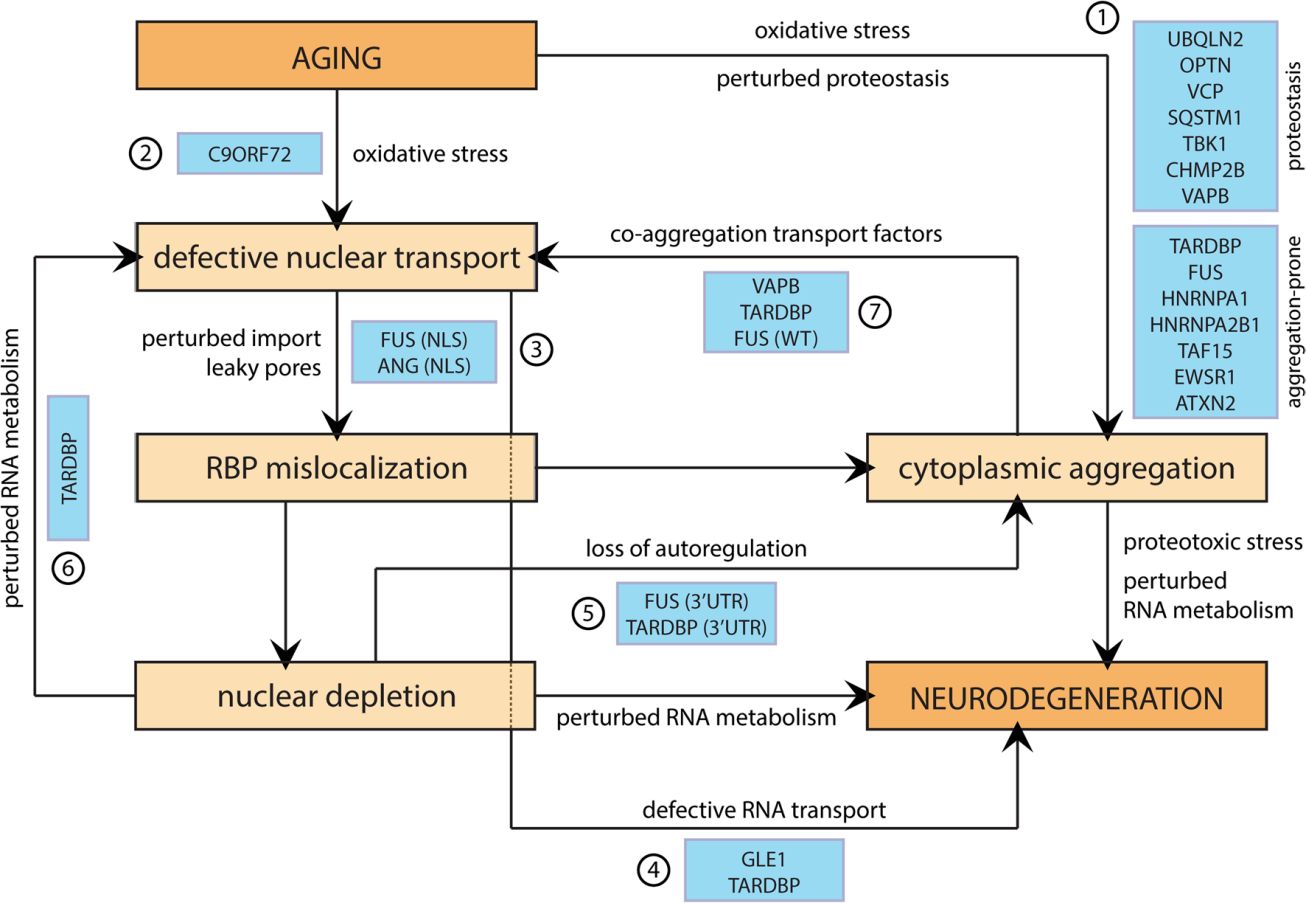
Roadmap to neuronal death in ALS/FTLD. Hypothetical scheme showing occurrence of different pathogenic events in RNA-binding protein-related ALS/FTLD. *Orange boxes* depict natural processes associated with aging. *Blue boxes* show where disease mutants enhance pathological pathways. See Supplementary Data for disease gene functions. *1* Numerous disease mutations make RNA-binding proteins more aggregation-prone or affect proteins involved in proteostasis [68, 98]. *2* *C9orf72* repeat expansions induce nucleocytoplasmic transport defects [8, 32, 48, 144]. *3* NLS mutants of both FUS [28] and Angiogenin (encoded by *ANG*) [93] have been reported, interfering with their proper nuclear targeting. *4* Mutations in GLE1 perturb RNA export [50], and mutant TDP-43 granules show altered axonal transport [2]. *5* 3′UTR mutants of FUS [147] and TDP-43 [14] interfere with their autoregulation resulting in overexpression of these proteins. *6* TDP-43 is an important genetic regulator of nuclear transport [106, 126]. *7* Various protein aggregates are known to sequester transport factors [91, 120, 130]

In this review, we tried to discuss all data currently available on the role of nucleocytoplasmic transport in ALS and FTLD. Moreover, we tried to address the question whether transport defects could be an initiating event in the disease. We used meta-analyses and bioinformatics to investigate this option. We found that nucleocytoplasmic transport cargoes are indeed associated with ALS/FTLD and related pathogenic processes. This suggests that a disruption of their proper localization could result in a loss of their normal functions, such as RNA splicing and transport, both vital in neuronal health. The function of RNA-binding proteins is strictly regulated, and largely depends on their subcellular localization. High levels of specific RNA-binding proteins in the cytoplasm will initiate spontaneous compartmentalization of these proteins into fluid-like granules, such as stress granules. These dynamic organelles are the prime suspects as seeds of the pathological aggregates. Compellingly, more than 70 % of all RNA-binding proteins misregulated in ALS/FTLD are known constituents of these stress granules. Recent evidence indeed suggests that RNA-binding proteins can undergo in vitro and in vivo concentration-dependent phase separations, and in vitro droplet maturation to pathological aggregates was observed in real time [79, 94]. These data highlight the need for a tight control of stress granule dynamics, with an important role for nuclear transport in this process. Hence, both loss of normal nuclear function and cytoplasmic aggregation of RNA-binding proteins seem to converge on nucleocytoplasmic transport. In Fig. 4, we provide an overview of how nuclear transport could fit in the framework of ALS/FTLD pathogenesis, and how disease mutants affect these processes.

The recent *C9orf72* modifier studies provided us with a list of interesting candidates which could be more broadly implicated in ALS and FTLD. Figuring out whether and how nucleocytoplasmic transport could be perturbed in sporadic patients will be one of the key future challenges. Nucleocytoplasmic transport has been therapeutically targeted before, but mostly by inhibition [40]. More fundamental research into the precise regulation of this process will be needed to find ways to boost, specifically, the affected nucleocytoplasmic transport pathways. Indeed, upregulation of this process has been shown to rescue neurodegeneration in yeast, fly and neuronal ALS/FTLD models [48, 126, 144]. Consequently, nucleocytoplasmic transport could become a novel and promising therapeutic target for ALS and FTLD.

## Electronic supplementary material

Below is the link to the electronic supplementary material.

Figure S1: Genes encoding RNA-binding proteins are implicated in ALS/FTLD pathology. Genes linked to ALS and FTLD [33, 99, 109, 110] were analyzed similarly as the pathology list. Network analysis using GeneMANIA indicates that these disease proteins are highly interactive. Physical interactions are depicted in red, genetic in green, and colocalization in blue. Disease genes could be roughly divided into six categories: ‘RNA metabolism’, ‘Nuclear transport’, ‘Cytoskeleton’, ‘Proteostasis’, ‘Vesicle transport’, and ‘Other’ (TIFF 81195 kb)

Figure S2: Modifiers of and pathological changes in ALS/FTLD models. **(a)** Overview of all genetic modifier studies in fly and yeast models of ALS/FTLD [4, 24, 32, 45, 48, 54, 113, 142, 144]. We filtered published dataset on proteins involved in the core process of nuclear transport (nuclear pore complex, importins, exportins, and Ran-GTP cycle) and included additional nuclear transport factors identified in the *C9orf72* studies. **(b)** Mislocalization and aggregation in cellular TDP-43 models as determined by SILAC quantitative mass spectrometry [107, 112] (TIFF 126736 kb)
